# Inhibition of human cytomegalovirus immediate-early gene expression and replication by the ethyl acetate (EtOAc) fraction of *Elaeocarpus sylvestris* in vitro

**DOI:** 10.1186/s12906-017-1941-7

**Published:** 2017-08-29

**Authors:** Sohee Bae, Se Chan Kang, Yoon-Jae Song

**Affiliations:** 1 Department of Life Science, Gachon University, Seongnam-Si, Gyeonggi-Do 13120 South Korea; 2 Department of Oriental Medicine Biotechnology, Kyung Hee University, Yongin, Gyeonggi-Do 17104 South Korea

**Keywords:** Human cytomegalovirus, Antiviral, *Elaeocarpus sylvestris*

## Abstract

**Background:**

In immunocompromised patients, human cytomegalovirus (HCMV) infection can lead to severe, life-threatening diseases, such as pneumonitis, hepatitis, gastrointestinal tract disease, and retinitis. We previously reported that a 70% ethanol extract of *Elaeocarpus sylvestris* leaves (ESE) inhibits human cytomegalovirus (HCMV) replication in vitro. In the present study, we determined the solvent fraction of ESE that inhibits HCMV replication using activity-guided fractionation.

**Methods:**

Activity-guided fractionation of ESE was performed to determine the solvent fraction that inhibits HCMV replication. Effects of solvent fractions on HCMV lytic gene expression and major immediate-early (MIE) enhancer/promoter activity were further investigated.

**Results:**

Among the solvent fractions tested, the EtOAc fraction of ESE markedly reduced HCMV lytic gene expression and viral replication in vitro without exerting significant cytotoxic effects against human foreskin fibroblasts (HFF). Furthermore, the EtOAc fraction negatively affected HCMV MIE enhancer/promoter activity.

**Conclusion:**

Our data collectively indicate that the EtOAc fraction of ESE contains active constituents that inhibit HCMV MIE enhancer/promoter activity and viral replication. The EtOAc fraction of ESE is a good source of novel drug candidates for treatment of HCMV-associated diseases.

## Background

Human cytomegalovirus (HCMV) is the largest human herpesvirus with a linear double-stranded DNA genome of about 230 kb encoding 167 viral proteins [1, 2]. HCMV can infect and replicate in several cell types including fibroblasts, neuronal cells, macrophages, dendritic cells, hepatocytes, epithelial cells and vascular endothelial cells. Through its wide cell tropism, the virus expands within the human body and between hosts [3, 4].

HCMV is distributed globally, infecting between 40 and 100% of the adult population worldwide [3]. Viral infection can spread through saliva, sexual contact, placental transfer, breastfeeding, blood transfusion or solid organ transplantation [1]. Primary HCMV infection induces immune responses, and the virus establishes latent infection [1]. In general, HCMV infection in normal children and adults is clinically asymptomatic [5]. However, HCMV infection in immunocompromised populations with damaged immunity can lead to severe, life-threatening diseases, such as pneumonitis, hepatitis, gastrointestinal tract disease, and retinitis [2, 6].

During lytic replication, the HCMV lytic gene is temporally expressed in an ordered cascade involving immediate-early (IE), early (E) and late (L) gene expression. The major IE1 and 2 genes encode important regulatory proteins, such as IE1-72 kDa and IE2-86 kDa that control early and late gene expression [7]. Early genes encode proteins that regulate viral DNA synthesis, while late genes encode proteins for virion components [2]. De novo viral protein synthesis is not required for the expression of viral IE genes. In combination with various cellular transcription factors, viral tegument proteins encoded by UL82 (pp71) and UL69 induce viral IE gene expression [8, 9].

Several agents, such as the nucleoside analog ganciclovir (GCV), nucleotide analog cidofovir (CDV) and pyrophosphate analog foscarnet (FOS), have been approved by the FDA for treatment of HCMV-related diseases [10]. The target of these synthetic drugs is viral DNA synthesis [2]. However, the usage of these synthetic drugs is associated with serious problems owing to several side-effects and the emergence of drug-resistant viruses. Previously, we determined the effects of a 70% ethanol extract of *Elaeocarpus sylvestris* leaves (ESE) on HCMV replication in vitro [11]. In the current investigation, we have focused on identifying the specific solvent fraction of ESE that inhibits HCMV replication in vitro and delineating the mechanism underlying anti-HCMV activity.

## Methods

### Cells, viruses and plant material

Maintenance and propagation of primary human foreskin fibroblasts (HFF), HEK293 cells and the Towne strain of HCMV (HCMV-Towne) have been described previously [12]. Plant materials (*Elaeocarpus sylvestris*) were collected from the Jeju Biodiversity Research Institute at Jeju island in Korea (specimen number JBR-083).

### Fractionation of ESE

**Fig. 1:**
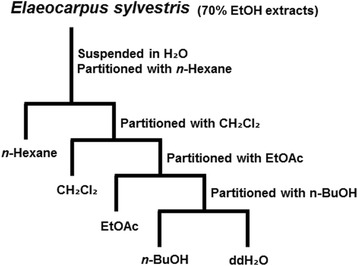
Fractionation scheme for ESE

*Elaeocarpus sylvestris* leaves were collected from Jeju island in Korea through Jeju Biodiversity Research Institute (Specimen number JBR-083). Dried *Elaeocarpus sylvestris* was exhaustively extracted with 70% ethanol (EtOH) twice at room temperature for 24 h. The ESE was concentrated under reduced pressure at 40 °C using a rotary evaporator to yield a semisolid dark-yellow residue. The extract was re-suspended in distilled water and successively fractionated using a series of solvents, including *n*-Hexane, dichloromethane (CH_2_Cl_2_), ethyl acetate (EtOAc), *n*-butanol (n-BuOH) and ddH_2_O (Fig. 1) [13].

### Quantification of HCMV via quantitative polymerase chain reaction (qPCR)

Isolation, amplification and quantification of HCMV DNA were performed as described previously [11].

### Plaque reduction assay

HFF cells were infected with serially diluted HCMV-Towne and treated with either DMSO or the EtOAc fraction at concentrations of 5, 10, 25 or 50 μg/ml. At 3 days after infection, cells were overlaid with 2 ml agar and 2 ml medium, and again at 10 days after infection. At 14 days after infection, cells were fixed with 10% formaldehyde at room temperature for 10 min, followed by staining with 0.03% methylene blue at room temperature for 5 min. The numbers of plaques were counted and expressed as plaque forming units per ml (pfu/ml).

### Western blot analysis

Cells were harvested, fractionated and transferred onto nitrocellulose membranes as described previously [14]. Antibodies to HCMV IE (IE1-72 kDa and IE2-86 kDa), ICP 36 and UL83 were purchased from Virusys (Taneytown, MD, USA). The anti-tubulin antibody was obtained from Sigma-Aldrich (St. Louis, MO, USA). Enhanced chemiluminescence detection reagent (Pierce, Rockford, Il.) and secondary peroxidase-labeled anti-mouse or anti-rabbit immunoglobulin G antibodies (Amersham Biosciences, Piscataway, NJ) were used according to the manufacturer’s recommendations.

### Cell viability assay

Cell viability was assessed using the CellTiter-Glo luminescent cell viability assay (Promega, WI), which determines the ATP levels in metabolically active cells, according to the manufacturer’s directions.

### Plasmid, transfections, and reporter gene assays

The reporter construct, pJHA324, containing HCMV MIE enhancer/promoter-driven firefly luciferase was generated from pCATwt760 and kindly provided by Dr. Jin-Hyun Ahn [15, 16]. NF-κB-dependent promoter-driven firefly luciferase was described previously [17]. OmisFect™ was used for transient transfection according to the manufacturer’s instructions (Omicsbio, Taipei City, Taiwan). Luciferase assays were performed as described previously [18].

### Statistical analysis

Statistical analyses were performed using JMP software (SAS Institute, Cary, NC). At least three independent experiments were conducted, and data were expressed as the mean ± standard deviation.

## Results

### Effects of ESE solvent fractions on HCMV replication

**Fig. 2:**
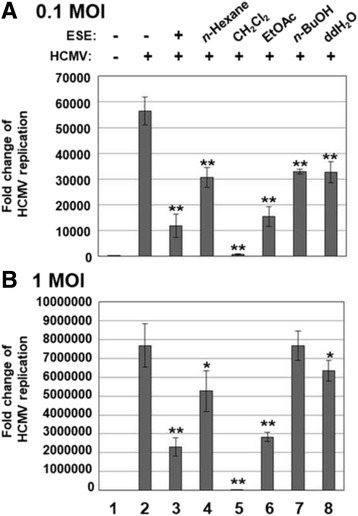
Effects of solvent fractions of ESE on HCMV replication. HFF cells were mock-infected (lane 1) or infected with HCMV-Towne at MOI of (**a**) 0.1 or (**b**) 1 (lanes 2 to 8) and treated with DMSO (lanes 1 and 2), ESE (lane 3) or *n*-Hexane (lane 4), CH_2_Cl_**2**_ (lane 5), EtOAc (lane 6), *n*-BuOH (lane 7) or ddH_2_O (lane 8) fractions of ESE at 50 μg/ml. At 3 days after infection, total DNA was harvested and the relative amounts of viral DNA were measured via qPCR using primers specific for UL123. Significant difference between samples was determined based on *P* values obtained from Student’s *t* test (* *P* < 0.01, ** *P* < 0.001)

ESE was fractionated via sequential solvent extraction, and the effects of individual fractions on HCMV replication was determined using qPCR with primers specific for UL123. At a low (0.1) multiplicity of infection (MOI), HCMV replication was reduced by each solvent fraction of ESE fractions at a concentration of 50 μg/ml (Fig. 2a). However, the EtOAC and CH_2_Cl_2_ fractions of ESE induced significant reduction of HCMV replication after infection at a high (1) MOI (Fig. 2b). The EtOAc fraction of ESE suppressed HCMV replication to a similar extent as ESE (Fig. 2a and b, compare lanes 6 and 3). Interestingly, the CH_2_Cl_2_ fraction of ESE exerted a stronger inhibitory effect against HCMV replication than ESE (Fig. 2a and b, compare lanes 5 and 3).

**Fig. 3:**
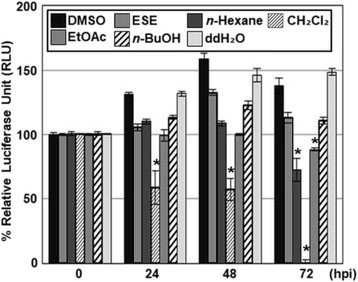
Cytotoxic effects of the fractions of ESE against HFF cells. HFF cells were treated with DMSO, ESE or the *n*-Hexane, CH_2_Cl_**2**_, EtOAc, *n*-BuOH or ddH_2_O fractions of ESE at 50 μg/ml, and viability was determined at 0, 24, 48 or 72 h after treatment using the CellTiter-Glo® Luminescent cell viability assay. Significant difference between samples was determined based on *P* values obtained from Student’s *t* test (* *P* < 0.01)

To ascertain whether the inhibitory effects of solvent fractions against HCMV replication are not related to cytotoxicity, HFF cells were treated with ESE solvent fractions at a concentration of 50 μg/ml, and cell viability was determined at 0, 24, 48 or 72 h after treatment using the CellTiter-Glo® Luminescent cell viability assay. Compared to 0 h, cellular ATP levels were elevated at 24, 48 and 72 h possibly due to active cellular metabolism and/or proliferation (Fig. 3). Surprisingly, the CH_2_Cl_2_ fraction of ESE induced a significant decrease in the viability of HFF cells (Fig. 3), suggesting that reduction of HCMV replication by the CH_2_Cl_2_ fraction of ESE is potentially mediated by its cytotoxicity. On the other hand, the EtOAc fraction of ESE did not exert significant cytotoxic effects against HFF cells until 48 h after treatment (Fig. 3). Compared to 0 h, the EtOAc fraction of ESE slightly reduced cellular ATP levels by 13% at 72 h after treatment (Fig. 3). Nevertheless, replenishment of the EtOAc fraction of ESE at 72 h exhibited no further effect on cellular ATP levels, and levels of cellular proteins including the p38 mitogen-activated protein kinase (p38 MAPK) and c-jun N-terminal kinase (JNK) were not attenuated by the treatment of the EtOAc fraction of ESE for 72 h (data not shown). Therefore, further experiments were conducted to elucidate the anti-HCMV activity and mechanism(s) of action of the EtOAc fraction.

### The EtOAc fraction of ESE inhibits HCMV replication in a dose-dependent manner

**Fig. 4:**
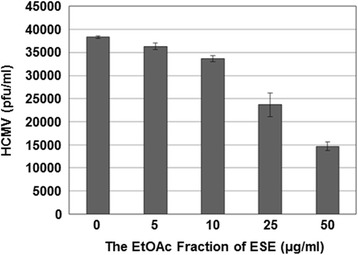
Dose-dependent suppression of HCMV replication by the EtOAc fraction of ESE. HFF cells were infected with serially diluted HCMV-Towne and treated with either DMSO or the EtOAc fraction of ESE at concentrations of 5, 10, 25 or 50 μg/ml. Cells were re-treated with DMSO or the EtOAc fraction of ESE every 3 days after infection. At 14 days following infection, the number of plaques was counted to determine pfu/ml using a plaque assay

To determine the inhibitory concentration of the EtOAc fraction of ESE that reduces the number of HCMV plaques by 50% (IC_50_), HFF cells were infected with serially diluted HCMV-Towne and treated with either DMSO or the EtOAc fraction of ESE at concentrations of 5, 10, 25 or 50 μg/ml. Cells were re-treated with DMSO or the EtOAc fraction every 3 days following infection, and the plaque reduction assay was performed to determine pfu/ml (Fig. 4). The IC_50_ value of the EtOAc fraction of ESE was estimated as 39 ± 2.65 μg/ml.

### The EtOAc fraction of ESE suppresses HCMV lytic gene expression

**Fig. 5:**
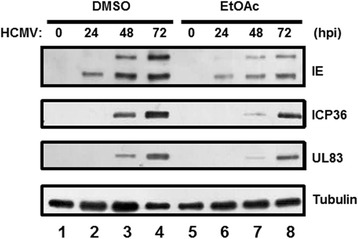
Downregulation of HCMV lytic gene expression by the EtOAc fraction of ESE. HFF cells were infected with HCMV-Towne at an MOI of 1 and treated with either DMSO or the EtOAC fraction of ESE (50 μg/ml). At 0, 24, 48 or 72 h after infection, equal amounts of cell extracts were subjected to western blot analysis with antibodies against HCMV IE (IE1-72 kDa and IE2-86 kDa), ICP 36, UL83 and tubulin

To determine the effects of the EtOAc fraction on HCMV lytic gene expression, HFF cells were infected with HCMV-Towne at an MOI of 1 and treated with either DMSO or the EtOAC fraction of ESE (50 μg/ml). At 0, 24, 48 or 72 h after infection, the levels of HCMV IE (IE1-72 kDa and IE2-86 kDa), E (infected cell protein 36, ICP 36) and L (UL83) proteins were determined via western blot (Fig. 5). In DMSO-treated cells, HCMV IE1-72 kDa protein was detected at 24 h after infection, and other lytic genes were strongly induced at 48 and 72 h after infection (Fig. 5, compare lane 1 with lanes 2 to 4). Notably, the EtOAc fraction induced a significant reduction in the levels of IE proteins and, in turn, E and L proteins (Fig. 5, compare lane 5 with lanes 6 to 8). The data collectively indicate that the EtOAc fraction of ESE contains an active constituent(s) that inhibits HCMV lytic gene expression and replication.

### The EtOAc fraction of ESE significantly reduces HCMV MIE enhancer/promoter activation

**Fig. 6:**
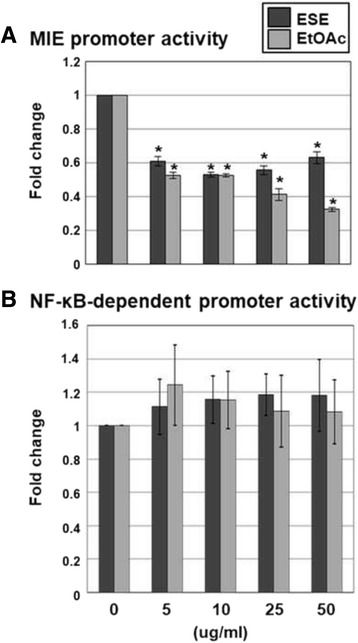
Effects of the EtOAc fraction of ESE on HCMV MIE enhancer/promoter. HEK293 cells were transfected with (**a**) HCMV MIE enhancer/promoter-driven firefly luciferase or (**b**) NF-κB-dependent promoter-driven firefly luciferase plus control *Renilla* luciferase plasmids. Cells were treated with DMSO, ESE or the EtOAC fraction of ESE at concentrations of 5, 10, 25 or 50 μg/ml, and luciferase activity was determined using a dual luciferase assay system. MIE enhancer/promoter- or NF-κB-dependent promoter-driven luciferase activity was expressed in RLU by normalizing firefly luciferase activity with constitutive *Renilla* luciferase activity. To calculate relative luciferase activity, MIE enhancer/promoter- or NF-κB-dependent promoter-driven firefly luciferase activity in the presence of DMSO was set as 1. Data represent the average of three independent experiments. (RLU, relative luciferase light unit) Significant difference between samples was determined based on *P* values obtained from Student’s *t* test (* *P* < 0.01)

In view of the finding that the EtOAc fraction of ESE disrupts HCMV IE protein expression, its effects on HCMV MIE enhancer/promoter activation were investigated. HEK293 cells were transfected with HCMV MIE enhancer/promoter-driven firefly luciferase and control *Renilla* luciferase plasmids and treated with either DMSO, ESE or the EtOAC fraction of ESE at concentrations of 5, 10, 25 or 50 μg/ml. At 5 and 10 μg/ml, both ESE and its EtOAc fraction reduced HCMV MIE enhancer/promoter activity by 40 and 48%, respectively (Fig. 6a). Interestingly, the EtOAc fraction of ESE exerted a stronger inhibitory effect on HCMV MIE enhancer/promoter activity than ESE and reduced activity in a dose-dependent manner (Fig. 6a). On the other hand, the EtOAc fraction of ESE did not exhibit an inhibitory effect on NF-κB-dependent promoter activity (Fig. 6b). Based on the results, we proposed that the EtOAc fraction reduces HCMV IE gene expression by down-regulating MIE enhancer/promoter activity.

## Discussion

Using solvent fractionation, we showed that the EtOAc fraction of ESE contains bioactive constituents that inhibit HCMV replication through downregulating MIE enhancer/promoter activation. Regulation of MIE enhancer/promoter activity is critical for HCMV latency, reactivation and pathogenesis [19]. Since the EtOAc fraction suppresses HCMV MIE enhancer/promoter activity in the absence of viral proteins, it may directly inhibit the function of cellular transcription factors or indirectly interfere with a signaling pathway(s) to activate a transcription factor(s) that regulates MIE enhancer/promoter activation. HCMV enhancer elements upstream of the MIE genes contain repetitive *cis*-acting sites that bind cellular transcription factors such as NF1, Elk-1, Sp-1, CAAT/enhancer binding protein, CREB/ATF, NF-κB, PAR/RXR and AP1 [19]. These transcription factors function cooperatively to bring the RNA polymerase II transcription initiation complex to the MIE promoter.

The biological function of ESE has not been established to date. ESE is reported to induce regeneration of hematopoietic cells, facilitate proliferation of lymphocytes and granulocytes and inhibit apoptosis in mice exposed to radiation [20, 21]. The chemical constituents of ESE are 2-hydroxy-benzaldehyde, coniferyl alcohol, umbelliferone, scopoletin, beta-sitosterol, daucosterol and 1,2,3,4,6-penta-O-galloyl-ß-D-glucose (PGG) [22]. Recently, we have reported that the EtOAc fraction of ESE contains luteolin-7-rutinoside, isoquercitrin, quercetin-3-O-arabinoside, luteolin-4-O-glucoside, quercetin, galloyl-D-glucose, di-galloyl glucose, gallic acid, digallic acid, tri-galloyl glucose, tetra-galloyl glucose, 1,2,3,4,6-penta-O-galloyl-ß-D-glucose and ellagic acid using the HPLC-Q-TOF-MS/MS analysis [23]. Among these compounds, PGG is highly enriched in numerous plants and exerts antiviral activities against various biological effects such as anti-cancer, anti-adipogenesis, anti-oxidative and anti-allergic activities [22].

Since AP1, a downstream transcription factor of the JNK pathway, is required for HCMV MIE enhancer/promoter activation, and the JNK inhibitor abolishes HCMV replication [24, 25], the EtOAc fraction of ESE may inhibit HCMV replication via disruption of JNK activation. Interestingly, PGG is reported to suppress the epidermal growth factor (EGF)-induced JNK activation [26]. Indeed, we found that PGG inhibits varicella-zoster virus (VZV) replication by interfering with VZV-induced JNK activation [23]. Accordingly, we hypothesized that PGG is potentially one of the active constituents in the EtOAc fraction of ESE with inhibitory effects on HCMV lytic gene expression and replication. However, we were unable to detect an inhibitory effect of PGG on HCMV MIE enhancer/promoter activity (data not shown). Examination of the possibility that a bioactive compound(s) in the EtOAc fraction additionally utilizes mechanisms other than JNK inhibition to suppress HCMV replication will be the focus of future studies.

## Conclusion

Our results demonstrate that the EtOAc fraction of ESE contains active compounds that interfere with HCMV MIE enhancer/promoter activation and replication. The EtOAc fraction of ESE is therefore a good source of novel drug candidates for treatment of HCMV-associated diseases, and the specific chemical constituents exerting anti-HCMV effects require further characterization.

## Abbreviations

ESE: Ethanol extract of *Elaeocarpus sylvestris*
HCMV: Human cytomegalovirus
HFF: Human Foreskin Fibroblast
IE: Immediate-early
